# Associations of Self-Reported Sleep Quality with Circulating Interferon Gamma-Inducible Protein 10, Interleukin 6, and High-Sensitivity C-Reactive Protein in Healthy Menopausal Women

**DOI:** 10.1371/journal.pone.0169216

**Published:** 2017-01-06

**Authors:** Wan-Yu Huang, Chih-Cheng Huang, Chia-Chu Chang, Chew-Teng Kor, Ting-Yu Chen, Hung-Ming Wu

**Affiliations:** 1 Institute of Basic Medical Sciences, College of Medicine, National Cheng Kung University, Tainan, Taiwan; 2 Department of Obstetrics & Gynecology, Changhua Christian Hospital, Changhua, Taiwan; 3 Department of Nephrology, Changhua Christian Hospital, Changhua, Taiwan; 4 Division of statistics, Internal Medicine Research Center, Changhua Christian Hospital, Changhua, Taiwan; 5 Inflammation Research & Drug Development Center, Changhua Christian Hospital, Changhua, Taiwan; 6 Department of Neurology, Changhua Christian Hospital, Changhua, Taiwan; 7 Graduate Institute of Acupuncture Science, China Medical University, Taichung, Taiwan; Katholieke Universiteit Leuven Rega Institute for Medical Research, BELGIUM

## Abstract

**Introduction:**

Sleep disturbance is very common in menopausal women and poor sleep quality has been linked to systemic inflammation. However, the impact of poor sleep quality on health outcomes of menopausal women remains unclear. This study evaluated the relationships between sleep quality and inflammation in menopausal women.

**Participants and design:**

This cross-sectional study enrolled 281 healthy women aged 45 to 60 years. The Pittsburgh Sleep Quality Index (PSQI) was used to measure quality of sleep. Multiplex assays were used to measure the levels of 9 cytokines in morning fasting plasma samples. Other variables measured in this study included clinical characteristics and high-sensitivity C-reactive protein (hs-CRP).

**Setting:**

The study was performed at a medical center.

**Results:**

The 281 participants comprised 79 (28%) perimenopausal women and 202 (72%) postmenopausal women. Global PSQI scores were positively correlated with plasma hs-CRP levels (*P* = 0.012) and were marginally associated with interferon gamma-inducible protein-10 (IP10), interleukin 6 (IL6), and macrophage inflammatory protein-1beta (MIP-1β) levels. After adjusting for age, body mass index, menopause duration, and follicle stimulating hormone, multiple linear regression analysis revealed that high PSQI scores and sleep efficiency < 65% were associated with elevated plasma levels of hs-CRP, IP10, and IL6. In addition, sleep duration < 5 hours was associated with high hs-CRP levels.

**Conclusion:**

Our data show that poor sleep quality and low sleep efficiency are associated with elevated levels of circulating inflammatory factors IP10, IL6 and hs-CRP and that short sleep duration is associated with high levels of hs-CRP in menopausal women. These findings provide novel evidence that poor sleep quality is linked to low-grade systemic inflammation in menopausal women.

## Introduction

Sleep disturbance is one of the core consequences of menopause. Menopausal women frequently complain of having difficulty in sleep initiation and/or sleep maintenance, and of early morning awakenings [1]. Many factors may play a role in menopausal sleep disturbance, mainly including hot flashes, sleep circadian rhythm changes, mood disorders, primary insomnia, co-existent medical problems and lifestyle factors [2–4]. The prevalence of sleep disturbance in middle-aged women increases with age and menopause stages [5]. A number of studies have shown that menopausal women are at higher risk of having sleep disturbance or insomnia than the general population [6].

Sleep influences a vast array of physiological functions such as immune function, cognitive ability, hypothalamic–pituitary–adrenal axis function (e.g. melatonin and cortisol secretion), and glucose metabolism [7]. For instance, the 24-hour sleep-wake cycle is involved in the regulation of the diurnal changes in immune cell number (e.g. neutrophils and macrophage) and levels of cytokines, which serve as chemical messengers to regulate immune cell behavior and response in blood [8,9]. When sleep is disturbed or restricted, disruption of the normal sleep-wake cycle can result in significantly increased levels of circulating innate immune cells such as granulocytes and monocytes, and impaired immunocyte function [10]. Moreover, the effects of sleep deprivation are cumulative. Over a period of time, sleep debt can lead to a wide range of deleterious health consequences including an increased risk of chronic systemic diseases such as hypertension and diabetes [11].

Increasing evidence indicates that insufficient sleep or sleep loss is strongly linked to. acute inflammation, which occurs over seconds to days, and chronic inflammation, which occurs over longer period of time and is usually low-grade and systemic [7,12]. A number of cytokines and chemokines, including interleukin-1 beta (IL-1β), tumor necrosis factor-alpha (TNF-α) and IL-6, as well as high-sensitivity C-reactive protein (hs-CRP) have been shown to be related to insomnia [10,13,14]. In addition, poor sleep quality and quantity have also been linked to systemic inflammation [15]. Although sleep disturbance is common in the menopausal population, few studies have examined the relationships between sleep disturbance and inflammation in menopausal women. Prather and colleagues reported that poor sleep quality, especially in combination with greater visceral adiposity, which is a risk factor for metabolic and cardiovascular diseases, was associated with stress-related increases in IL6 levels and IL6/IL10 ratio in postmenopausal women [16]. In addition, Prinz and colleagues reported that increased plasma IL1β and cortisol levels were associated with impaired sleep quality in senior women (mean age, 70.6 ± 6.2 years) [17].

Sleep plays an essential role in the regulation of innate and adaptive immune response [7]. We hypothesized that poor sleep quality is linked to systemic inflammation in menopausal women. Therefore, this study investigated the relationships between self-reported sleep quality and systemic inflammation in menopausal women. Sleep quality was evaluated with the Pittsburgh Sleep Quality Index (PSQI) and inflammatory status was determined by measuring the levels of 9 cytokines/chemokines using multiplex assays as well as by measuring hs-CRP levels in healthy menopausal women.

## Subjects and Methods

### Participants and study design

The subjects in this cross-sectional study comprised women aged 45 to 60 years who visited the Changhua Christian Hospital for health management reasons during the period January 2013 to January 2016. Women were eligible for inclusion if they had experienced alterations in menstrual frequency and/or flow in the past 12 months (perimenopause) and had at least 12 consecutive months of amenorrhea (postmenopause) not due to surgery or other obvious causes preceding entry into the study. Women were excluded if they were hormone therapy users, had undergone hysterectomy or bilateral oophorectomy, or had a history of diabetes, hypertension, or thyroid disease. The patient records and information were anonymized and de-identified prior to analysis. Written informed consent was obtained from all participants. This study was approved by the Changhua Christian Hospital Institutional Review Board (ID: CCH IRB No. 110305).

### Anthropometric measures

Venous blood samples were collected from participants between 08:00–10:00 h after an overnight fast. The plasma was aliquoted and stored at −80°C without thawing until assay. Height and weight were measured in light clothing without shoes. Body mass index (BMI) was calculated as weight (kg)/height (m)^2^.

### Self-reported sleep quality

The Pittsburgh Sleep Quality Index (PSQI) is a widely used and reliable measure of sleep quality [18]. The Taiwanese version of the PSQI used in the present study has been demonstrated to be a reliable, valid, and sensitive instrument to measure sleep quality in Taiwanese [19]. The index is a 19-item self-rated questionnaire that measures sleep disturbance along seven dimensions: subjective sleep quality, sleep latency, sleep duration, habitual sleep efficiency, sleep disturbance, use of sleeping medications, and daytime dysfunction over the last month. Each dimension is rated on a four-point Likert scale and the scores from these dimensions are added together to generate a global score ranging from 0 to 21. Participants were asked to complete the self-rated PSQI questionnaire to determine sleep quality during the month prior to study entry. Sleep duration was divided into four scales: > 7 hours of sleep each night (scale 0), 6–7 hours (scale 1), 5–6 hours (scale 2), and < 5 hours (scale 3). Sleep efficiency, defined as the ratio of total time spent asleep to the total amount of time spent in bed, was divided into four scales: ≥ 85% (scale 0), < 85% but > 75% (scale 1), < 75% but > 65% (scale 2), and < 65% (scale 3).

### Measurements of follicle-stimulating hormone and high-sensitivity C-reactive protein

Serum fasting glucose, high-sensitivity C-reactive protein (hs-CRP), and follicle-stimulating hormone (FSH) were measured using standard procedures at the Department of Laboratory Medicine, Changhua Christian Hospital.

### Measurements of plasma cytokines and chemokines

The plasma levels of chemokine CXCL10 also known as interferon-inducible protein-10 (IP10), chemokine CCL2 also known as monocyte chemoattractant protein-1 (MCP-1), and chemokine CCL4 also known as macrophage inflammatory protein-1beta (MIP-1β) were measured using a Millipore cytokine three-plex panel assay (MILLIPLEX MAP Human Cytokine/Chemokine Magnetic Bead Panel)(Milliplex MAP kits, EMD Millipore, Billerica, MA, USA). The plasma levels of interferon gamma (IFNγ), tumor necrosis factor-alpha (TNF-α), interleukin-beta (IL-β), IL6, chemokine CXCL8 also known as IL8, and IL17A were determined using a Millipore cytokine seven-plex panel assay (MILLIPLEX MAP Human Cytokine/Chemokine Magnetic Bead Panel) (Milliplex MAP kits, EMD Millipore, Billerica, MA, USA). All analyses were performed by T.Y. Chen according to the manufacturer’s protocol. The results were read using a Luminex 200 system (Luminex, Austin, TX, USA). Values of these cytokines and chemokines were reported as pg/ml. Data on cytokines and chemokines were collected and analyzed using an instrument equipped with MILLIPLEX Analyst software (EMD Millipore). For these 9 cytokines, the intra-assay laboratory coefficients of variation were less than 8% and the inter-assay coefficients of variation were less than 10%.

### Statistical analysis

Data are presented as median (IQR: interquartile range) or percentage. Variables were tested for normal distribution using the Kolmogorov-Smirnov test. Pearson’s correlation analysis was performed to examine the association between global PSQI scores and each of the 9 cytokines/chemokines and hs-CRP. Significant variables at the 0.1 level were included in a regression model. After log-transformation (ln) of non-normally distributed variables, univariate and multivariate linear regression analyses were performed to determine the association between sleep quality measures and each of these variables, and the results are represented as standardized coefficients (beta). In the multivariate analysis, the model was adjusted for the traditional confounders age, menopause duration, and BMI, and for the non-traditional possible confounder FSH [20,21]. A two-tailed *P* value < 0.05 was considered to be statistically significant. Statistical analyses were performed with SPSS software (Version 19.0.0, IBM Corporation, Somers, NY, USA).

## Results

**Fig 1:**
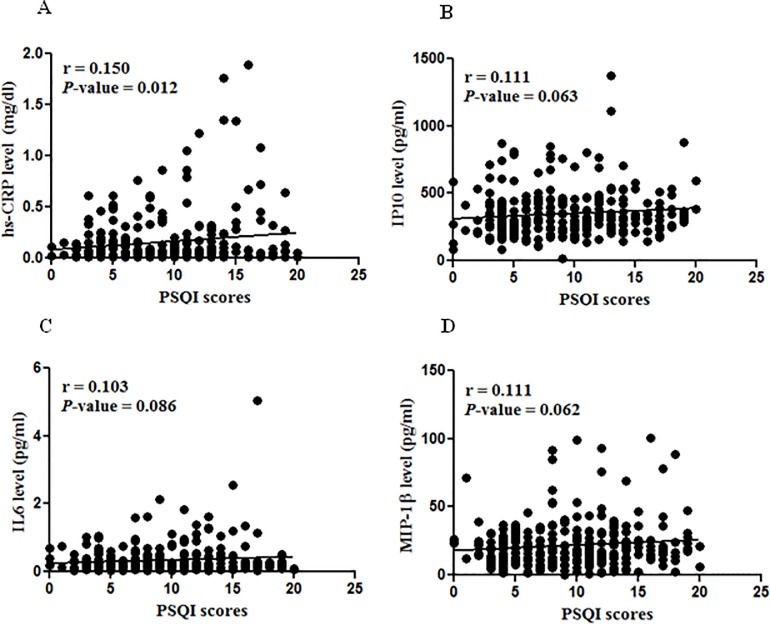
Correlation between PSQI scores and inflammatory factors. Pittsburgh Sleep Quality Index (PSQI) scores correlated with high-sensitivity C-reactive protein (hs-CRP) (A), interferon-inducible protein-10 (IP-10) (B), interleukin 6 (IL6) (C), and macrophage inflammatory protein-1beta (MIP-1β) (D) at *P* values less than 0.1 using Pearson’s correlation analysis.

A total of 281 women, including 79 (28%) perimenopausal women and 202 (72%) postmenopausal women, fulfilled the entry criteria and were enrolled in this cross-sectional study. Table 1 shows the clinical baseline characteristics, circulating cytokine/chemokine levels, and sleep quality measures of the participants. We examined the overall correlation between sleep quality and each of the 9 cytokine/chemokines as well as hs-CRP using Pearson’s correlation analysis. As shown in Fig 1, four factors were revealed to be significantly associated with PSQI scores at *P* < 0.1, namely hs-CRP (*P* = 0.012), IP10 (*P* = 0.063), IL6 (*P* = 0.086), and MIP-1β (*P* = 0.062). These four factors were therefore selected for regression analysis. Multivariate regression analysis revealed that PSQI scores were significantly associated with plasma levels of hs-CRP (*P* < 0.01), IP10 (*P* < 0.05), and IL6 (*P* < 0.05) after adjusting for the confounders age, BMI, menopause duration and FSH (Table 2). However, there was no significant association between PSQI scores and MIP-1β.

**Table 1:** Baseline characteristics of the participants in this study.

Variables	Total (n = 281)
**Participant characteristics**	
Perimenopause, n (%)	79(28)
Postmenopasue, n (%)	202(72)
Age, year	54(51,56)
MP_duration, year	3(1,6)
BMI, kg/m^2^	23.4(21.5,25.6)
FSH, mIU/ml	61.9(35,78)
**Global and component scores of PSQI**	
Global PSQI scores	9(5,13)
Sleep latency scores	2(1,3)
Sleep duration scores	2(1,2)
Sleep efficiency scores	1(0,2)
Sleep quality scores	2(1,2)
Sleep maintenance scores	1(1,2)
Medication for sleep scores	0(0,3)
Daytime dysfunction scores	0(0,2)
**Inflammatory measures**	
hs-CRP, mg/dl	0.058(0.016,0.176)
IFNr, pg/ml	3.36(1.51,6.39)
IL17A, pg/ml	1.02(0.69,2.001)
IL1β, pg/ml	0.588(0.44,0.896)
IL6, pg/ml	0.195(0.112,0.39)
IL8, pg/ml	0.82(0.63,1.204)
TNFα, pg/ml	0.34(0.2,0.75)
IP10, pg/ml	323(254,431)
MCP-1, pg/ml	281(230,352)
MIP-1β, pg/ml	18.3(11.28,30)

Data are presented as median (Q1, Q3) or n (percentage).

Abbreviations: Q1, Quartile 1 (25^th^ percentile); Q3, Quartile 3 (75^th^ percentile); PSQI, Pittsburgh Sleep Quality Index; MP_duration, menopause period since final menstrual period; BMI, body mass index; FSH, follicle stimulating hormone; hs-CRP, high-sensitivity C-reactive protein; IFNγ, interferon gamma; TNFα, tumor necrosis factor alpha; IL1β, interleukin one beta; IL6, interleukin 6; IL8, interleukin 8; IL17A, interleukin 17A; IP10, interferon-inducible protein-10; MCP-1, monocyte chemoattractant protein-1; MIP-1β, macrophage inflammatory protein-1beta.

**Table 2:** Associations between sleep quality and inflammatory factors.

Variables	Sample size	CRP		IP-10			IL-6		MIP-1β	
		unadjusted	adjusted	unadjusted		adjusted	unadjusted	adjusted	unadjusted	adjusted
**Global PSQI**										
PSQI	281	4.91	5.21	1.12	1.24		2.79	3.77	1.84	1.81
		(1.1,8.9)^a^	(1.44,9.12)^b^	(-0.1,2.3)	(0.1,2.4)^a^		(-0.4,6.1)	(0.75,6.89)^a^	(-0.1,3.8)	(-0.1,3.7)
**Components of PSQI**										
**Sleep efficiency**										
0	72	1	1	1	1		1	1	1	1
1	102	58.94	60.34	4.72	8.58		-2.89	-7.32	-12.59	-14.17
		(-23.9,231.8)	(-21.3,226.8)	(-9.9,21.76)	(-6.2,25.7)		(-38.1,52.3)	(-40.1,43.4)	(-32.0,12.4)	(-32.6,9.3)
2	54	82.9	104.16	-3.38	0.26		-7.8	-16.85	14.76	10.79
		(-15.3,294.9)	(-2.1,325.87)	(-19.5,16.0)	(-16.1,19.8)		(-46.6,59.0)	(-52.4,45.3)	(-11.3,48.4)	(-13.7,42.3)
3	53	181.62	179.23	25.78	28.72		76.19	76.92	19.74	15.14
		(39.4,469.6)^b^	(40.1,456.4)^b^	(7.2,47.5)^b^	(9.9,50.7)^b^		(17.2,164.9)^b^	(21.4,157.8)^b^	(-7.0,54.2)	(-10.1,47.5)
**Sleep duration**										
0	39	1	1	1	1		1	1	1	1
1	79	12.85	34.82	-4.7	-3.86		-8.77	-24.42	-14.89	-19.24
		(-55.3,184.7)	(-48.8,255.1)	(-21.515.7)	(-20.4,16.2)		(-47.0,57.2)	(-57.1,33.1)	(-37.4,15.6)	(-40.1,8.9)
2	97	44.91	67.76	2.13	2.32		1.4	-0.25	-3.53	-7.16
		(-38.5,241.6)	(-32.1,314.6)	(-14.9,22.7)	(-14.3,22.2)		(-38.9,68.5)	(-38.9,62.9)	(-27.3,28.0)	(-28.8,21.2)
3	66	140.71	184.72	15.26	16.44		17.94	14.03	11.89	4.66
		(5.5,449.0)^a^	(16.8,594.3)^a^	(-4.4,39.0)	(-3.3,40.1)		(-29.5,97.3)	(-30.5,87.2)	(-16.0,49.1)	(-20.9,38.5)

Data are expressed as the percentage difference (95% CI).

Regression coefficients are back-transformed using formula (100*(exp(β)-1)) to calculate the percentage difference and the 95% CI in IP10, IL6, MIP-1β, and hs-CRP levels for global PSQI scores, sleep duration categories, or sleep efficiency categories per 1 unit increment.

Linear regression model was adjusted for age, menopause duration, body mass index and follicle stimulating hormone.

Sleep duration and sleep efficiency were categorized into four scales scored from 0 to 3, respectively.

Abbreviations: PSQI, Pittsburgh Sleep Quality Index; hs-CRP, high-sensitivity C-reactive protein; IL6, interleukin 6; IP10, interferon-inducible protein-10; MIP-1β, macrophage inflammatory protein-1beta.

a, *P*-value < 0.05

b, *P*-value < 0.01

Habitual sleep duration and sleep efficiency have been shown to be associated with a pro-inflammatory state [22]. In the present study, we also examined the relationships of these two components of sleep quality with the selected inflammatory factors. Sleep duration and sleep efficiency were categorized into four scales, respectively. A total of 39 participants had a sleep duration scale of 0, 79 had a scale of 1, 97 had a scale of 2 and 66 had a scale of 3. We found that sleep duration < 5 hours (scale 3) was significantly associated with elevated plasma levels of hs-CRP (*P* < 0.05) after adjusting for age, menopause duration, FSH, and BMI. In addition, a total of 72 women had a sleep efficiency scale of 0, 102 had a scale of 1, 54 had a scale of 2, and 53 had a scale of 3. A sleep efficiency of < 65% (scale 3) was significantly associated with circulating levels of hs-CRP, IP10, and IL6 (all *P* values < 0.01) (Table 2).

## Discussion

In this investigation of the relationship between sleep quality and inflammation in menopausal women, we minimized the potential confounding factors by controlling for a number of variables that are linked to systemic inflammation (e.g. diabetes [23], hypertension [24,25], and smoking [26]). We found that poor sleep quality, as determined by high PSQI scores and a sleep efficiency scale of < 65%, was associated with elevated plasma levels of the pro-inflammatory factors IP10 and IL6 and hs-CRP. In addition, sleep duration < 5 hours was significantly associated with elevated hs-CRP concentrations. These results suggest that poor sleep quality is linked to low-grade systemic inflammation in menopausal women.

Central sleeping/waking circadian rhythms are associated with changes in peripheral cytokine expression and cellular immune functions [14,27]. Disrupted sleep will logically alter their expression and bioactivity. Previous studies have reported that sleep deprivation and poor sleep quality induce changes in a number of circulating pro-inflammatory cytokines [28,29]. Cumulative evidence further shows that inflammation status is related to the patterns of sleep disturbance such as sleep duration and efficiency as well as by gender, age, and comorbid diseases [29]. Hs-CRP has been demonstrated to be a very sensitive measure of systemic inflammation Recently, several large epidemiological and clinical studies have explored the association between poor sleep quality and hs-CRP [15,30]. Liu and colleagues found that poor sleep quality was associated with elevated plasma levels of hs-CRP in females but not in males in a U.S. adult population [15]. The present study also detected a consistent association between poor global sleep quality and circulating hs-CRP concentrations in menopausal women [15,16]. Among the night pro-inflammatory factors measured in the study, we found the IL6 and IP10 are also manifestations of poor global sleep quality and poor sleep efficiency in healthy menopausal women [12].

IP-10 is a chemokine produced by a wide variety of cells, including hepatocytes, endothelial cells, neutrophils, monocytes, splenocytes, activated T lymphocytes, and astrocytes [31]. IP10 has been shown to play a key role as an inflammatory mediator in several diseases including viral infections (e.g. hepatitis C), diabetes, cardiovascular disease, nonalcoholic fatty liver disease, and neurodegenerative diseases [32–34]. Few studies, however, reported the relationships between IP10 and sleep disturbance. Jain et al found that IP10 levels were elevated in type 2 diabetes patients with insomnia, sleep apnea or both [35]. We found that IP10 in healthy menopausal women was significantly related to poor sleep quality as well as low sleep efficiency. These results suggest that IP10 is also a potential marker of systemic inflammation for poor sleep quality in menopausal women, although it needs more studies to support the findings.

Habitual sleep efficiency in this study was defined as the ratio of sleep time to total time in bed. Many factors may affect sleep efficiency, such as sleep disorders (e.g. insomnia), restless leg syndrome and social engagement [3,4]. IL6 expression relative to sleep has well characterized diurnal changes [28]. Normally, IL6 concentrations are increased in the beginning of falling asleep and highest at night and decreased in daytime. Disturbed nocturnal sleep will destroy this IL6 rhythm, leading to daytime overexpression. Several studies reported poor habitual sleep efficiency were associated elevated levels of morning IL6 levels [4,36]. This relationship was further demonstrated by objective measurement with polysomnography to be related to disturbed patterns of sleep/wake schedule such as increased latency of rapid eye movement (REM) and increased portion of wake after sleep onset (WASO) [36,37]. In healthy menopausal women, the present study found that the association of poor sleep efficiency was consistent with IL6, but also with IP10 and hs-CRP.

Altered sleep duration may be related to adverse health outcomes. The mechanisms remain unclear but could be related to increased inflammation. Several studies have revealed that IL6 may play a role in habitual sleep duration on inflammation [38,39]. For instance, elevated IL6 levels are associated with shorter (e.g. < 5 hrs) and longer (e.g. > 8 hrs) sleep duration. That is, the association between IL6 and sleep duration categories possibly follows a U-shaped trend. However, IL6 and IP10 were inconsistently associated with short sleep duration in our study. It is possible that the category for longer sleep duration (e.g. > 8 hours) was not identified from the category > 7 hours in this study. In addition, the small sample size in our study did not allow for the detection of significant differences between the categories of sleep duration. In contrast to the results of the cytokines (e.g. IL6), which may be affected by circadian changes, circulating CRP appears to be stable over long periods of time [40]. Our study found that elevated levels of hs-CRP are closely associated with short sleep duration in the healthy menopausal women, but a number of studies have shown inconsistent results regarding the association between sleep duration and hs-CRP levels in the other populations [41–44]. For instance, the findings from the Wisconsin Sleep Cohort study and the National Health and Nutrition Examination Survey (NHANES) failed to support any significant association between short sleep duration and hs-CRP [41,42]. Jackowska et al. found that long sleep duration was associated with increased CRP only in men [43], and Miller et al. found that short sleep duration was linked to high hs-CRP levels only in women [44]. Clearly, to confirm the effects of poor sleep quality on these inflammatory factors, there is a need for further studies with a larger sample size and a more ideal design, particularly for sleep duration with the cutpoints used to precisely define shorter and longer categories, for sleep efficiency with detailed information of individual sleep habit and objective assessment such as polysomnography or actigraphy [45].

IP10, IL6 and hs-CRP have been demonstrated to be risk factors for metabolic and cardiovascular diseases [35,46,47]. Epidemiological studies consistently demonstrate associations between suboptimal sleep and systemic chronic diseases such as coronary heart disease, stroke, and diabetes [43,48,49]. Our findings show that the sleep disturbance-associated inflammatory factor IP10 as well as IL6 and hs-CRP may indicate an underlying inflammatory state, which has been shown to be associated with increased risk for cardiovascular disease in menopausal women [50]. Although exactly how biochemical processes cause inflammation in women with poor sleep quality is not yet understood, the mechanisms of sleep disturbance in menopause are known to include vasomotor symptoms, circadian rhythm factor, primary insomnia, psychosocial factor, and mood factor [4, 2,3]. These contributing factors have been reported to have close linkage to systemic inflammation [7,51]. Furthermore, Friedman successfully demonstrated that positive psychological engagement may compensate for the elevated levels of IL6 associated with poor sleep quality, and for biological risk for disease [4]. Therefore, further studies are needed to validate the link between these potential inflammatory biomarkers demonstrated in our study and in previous studies and sleep disturbance. The results may provide evidence that can be used to develop a therapeutic strategy to reduce the impact of sleep quality on inflammation in menopausal women.

There are several limitations that need to be addressed. First, this investigation is not sufficient to determine whether a causal relationship exists between IP10, IL6, and hs-CRP and poor sleep quality because it was a cross-sectional study. Second, the number (n = 202) of postmenopausal women in this study was markedly higher than that (n = 79) of perimenopausal women. The imbalance in sample sizes may affect the statistic validity, although there were no significant differences in PSQI scores or levels of inflammatory cytokines/chemokines between the two groups. Third, the small sample size (n = 281) may have increased the risk of making a Type II error, which is one possibility to explain why two inflammatory factors IL6 and IP10 appear inconsistent across sleep duration outcomes. Fourth, multiplex techniques employing proprietary bead sets offer the potential of measuring multiple cytokines in the same sample simultaneously. However, the multiplex techniques used for cytokines/chemokines assays could not avoid the influence of the soluble receptor binding proteins for cytokines (e.g. IL6) [52]. The possibility may result in an underestimation of the cytokine levels in our study. Although the hypersensitivity assay for CRP has been well-established and has been shown to be suitable for routine clinical routine use [53,54], while the accuracy of hs-CRP levels can be compromised by patient with potential upper respiratory infection, although all of our participants were apparently healthy. Finally, the assessment of sleep quality was limited because we used the PSQI self-report questionnaire to assess sleep disturbance, sleep duration and sleep efficiency. Herbal medicine and acupuncture is commonly used in Taiwan for insomnia and sleep habits often vary by individuals. Lack of this detailed information from our participants may have led to potential insensitivities of the PSQI instrument for sleep quality assessment.

In summary, we found that poor sleep quality and low sleep efficiency were significantly associated with elevated levels of circulating hs-CRP, IP10 and IL6 while short habitual sleep duration was highly related to high hs-CRP levels in menopausal women. These findings provide novel evidence that low-grade inflammation plays an important role in poor sleep quality in healthy menopausal women. Further longitudinal studies are required to clarify the causal relationships between these inflammatory factors and sleep disturbance in menopausal women.
